# Serial Section-Based Three-Dimensional Reconstruction of *Anaxagorea* (Annonaceae) Carpel Vasculature and Implications for the Morphological Relationship between the Carpel and the Ovule

**DOI:** 10.3390/plants10102221

**Published:** 2021-10-19

**Authors:** Ya Li, Wei Du, Ye Chen, Shuai Wang, Xiao-Fan Wang

**Affiliations:** 1 College of Life Sciences, Wuhan University, Wuhan 430072, China; liavanilla@whu.edu.cn (Y.L.); wdu@whu.edu.cn (W.D.); 2 Department of Environmental Art Design, Tianjin Arts and Crafts Vocational College, Tianjin 300250, China; chenye891130@icloud.com; 3 College of Life Sciences and Environment, Hengyang Normal University, Hengyang 421001, China; handsome_king@whu.edu.cn

**Keywords:** anatomy, 3D reconstruction, *Anaxagorea*, angiosperms, carpel, organogenesis, vascular system

## Abstract

Elucidating the origin of flowers has been a challenge in botany for a long time. One of the central questions surrounding the origin of flowers is how to interpret the carpel, especially the relationship between the phyllome part (carpel wall) and the ovule. Recently, consensus favors the carpel originating from the fusion of an ovule-bearing part and the phyllome part that subtends it. Considering the carpel is a complex organ, the accurate presentation of the anatomical structure of the carpel is necessary for resolving this question. *Anaxagorea* is the most basal genus in a primitive angiosperm family, Annonaceae. The conspicuous stipe at the base of each carpel makes it an ideal material for exploring the histological relationships among the receptacle, the carpel, and the ovule. In the present study, floral organogenesis and vasculature were delineated in *Anaxagorea luzonensis* and *Anaxagorea javanica*, and a three-dimensional model of the carpel vasculature was reconstructed based on serial sections. The results show that in *Anaxagorea*, the vasculature in the carpel branches in the form of shoots. The radiosymmetrical vasculature pattern is repeatedly presented in the receptacle, the carpel, and the funiculus of the ovule. This provides anatomical evidence of the composite origin of the carpel.

## 1. Introduction

Since Darwin’s time, the elucidation of the origin and evolution of flowering plants has been a primary goal of plant science [1]. In the origin of flowers, the emergence of carpels was a key innovation. In the evolutionary origin of angiosperms, the emergence of carpel was the first step, followed by double fertilization and the emergence of flowers [2]. The carpel protectively encloses the ovules and sets angiosperms apart from other seed plants, which develop exposed ovules [3,4,5]. Despite ovules being part of carpels and enclosed in carpels in all living angiosperms, there are phylogenetic indications that carpel and ovule were different morphological units at their evolutionary beginnings [6,7,8,9,10,11].

Therefore, how to explain the relationship between carpel and ovule is of significance to the origin of flowers. The carpel might have evolved by synorganization, involving a recurved uniovulate cupule and a subtending bract or leaf [12]. At present, many studies have attempted to discuss the relationship between angiosperm carpel and ovule from phylogeny, molecular biology, and ontogeny perspectives [13,14,15,16,17,18,19]. However, associated anatomical studies are few [20,21,22]. Considering the carpel is a complex organ, more accurate methods are required for the description of the actual anatomical structure of carpels, besides discontinuous sections and line drawings.

In the present study, *Anaxagorea* was selected for floral organogenesis and vascular anatomic examination. *Anaxagorea* is the most basal genus in Annonaceae, which represents one of the largest families in the Magnoliales, and is one of the most important lineages in the early radiation of angiosperms [23,24,25,26,27]. The carpels of *Anaxagorea* are apocarpous (free) throughout their life history [28], and each has a notably long carpel stipe [29]. Two species were collected for the study: *Anaxagorea luzonensis* from Hainan and *Anaxagorea javanica* from Yunnan. The aim of the present study was to obtain an accurate anatomical structure model of the carpel, and histologically analyze relationships among the receptacle, the carpel, and the ovule, based on vasculature through continuous anatomical observations and three-dimensional (3D) reconstruction, so as to provide an anatomical basis for interpreting the relationship between the carpel and the ovule.

## 2. Results

### 2.1. Gynoecium Structure and Carpel Organogenesis

**Figure 1:**
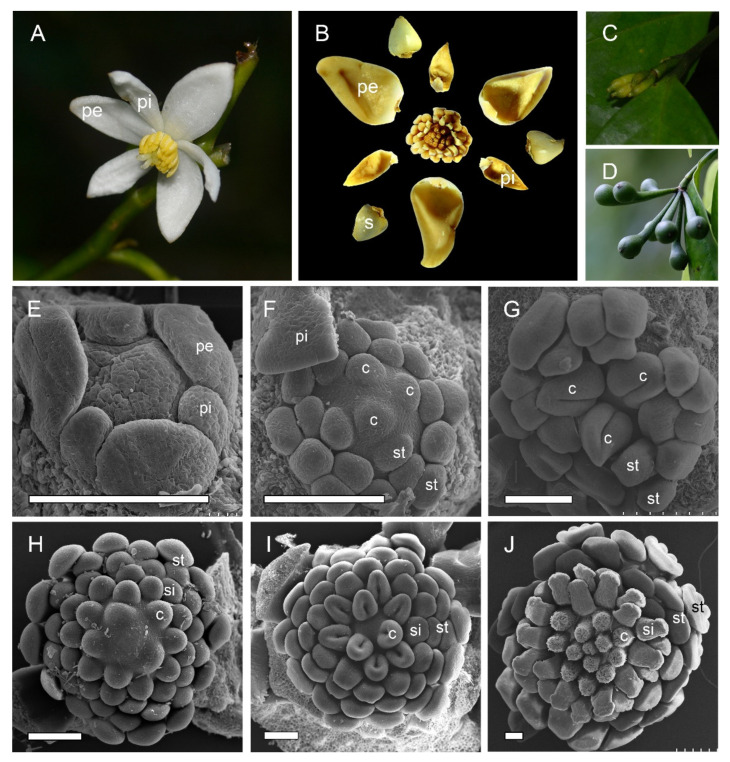
Floral morphology and gynoecium development in two *Anaxagorea* species. (**A**) *Anaxagorea luzonensis* flower. (**B**) *Anaxagorea javanica* flower. (**C**) Young *A. luzonensis* fruit. (**D**) Mature *A. javanica* fruit. (**E**–**G**) *A. luzonensis* floral development. (**H**–**J**) *A. javanica* gynoecium development. c, carpel; pe, outer petal; pi, inner petal; s, sepal; si, staminode; st, stamen. Scale bars = 200 μm.

The flowers of two study species were trimerous with a whorl of sepals, two morphologically distinct whorls of petals, and numerous stamens (and inner staminodes of *A. Javanica*) (Figure 1A–D).

**Figure 2:**
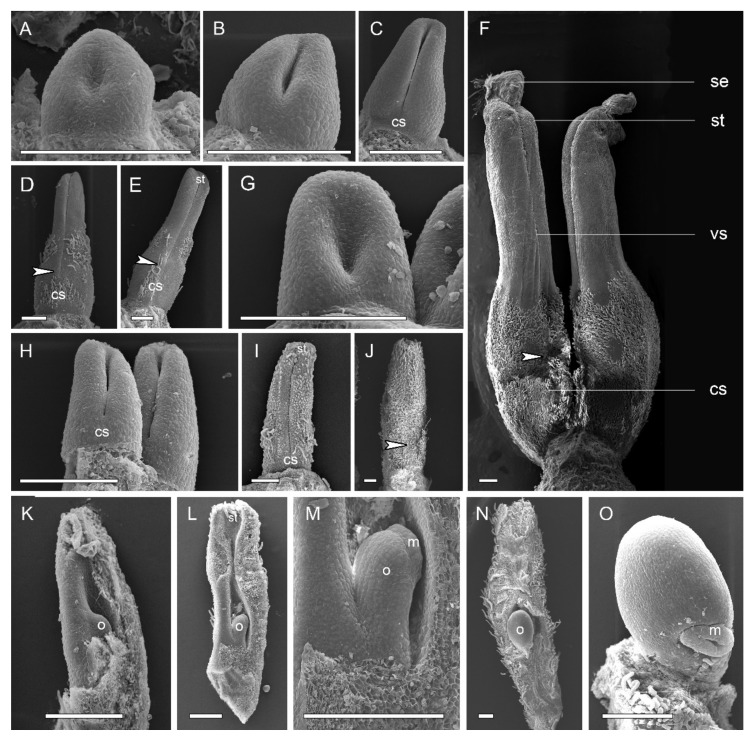
Carpel organogenesis in two *Anaxagorea* species. (**A**–**F**) *Anaxagorea luzonensis*. (**A**) Carpel primordia. (**B**,**C**) Carpel stipe emergence. (**D**,**E**) Carpel thickening and stigma formation, showing carpel stipe elongation. (**F**) Mature carpels. (**G**–**O**) *Anaxagorea javanica* shows similar carpel developmental features to changes depicted in **A**–**F**. Ventral slit end indicated by arrows. (**K**) The ovule before the integuments initiate. (**L**,**M**) Young ovule, with the carpel at the stage of **I**. (**N**,**O**) The ovule nearly mature. Cs, carpel stipe; m, micropyle; o, ovule; se, stigmatic exudate; st, stigma; vs, ventral slit. Scale bars = 200 μm.

**Figure 3:**
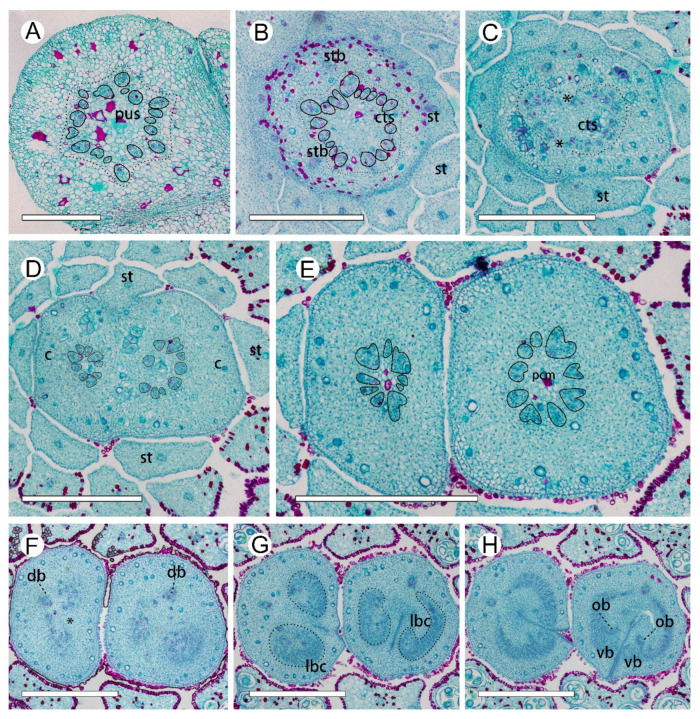
Ascending paraffin transections of *Anaxagorea luzonensis* flower. (**A**) Base of receptacle, showing the pseudostele (dotted line). (**B**) Mid-section of androecia, showing stamen bundles and central stele (dotted line). (**C**) Top of receptacle, showing central stele divided into two groups (* marked the breaks). (**D**) Bundles from the central stele enter carpels (each bundle is marked by a solid line). (**E**) Base of carpels, showing basal ring. (**F**) Upper part of carpel stipes, showing the basal ring breaks (marked as *). (**G**) Bottom of ovary locule, showing concentric lateral bundle complexes (left) and “C”-shaped lateral bundle complexes (right). (**H**) Base of ovary locule. Cts, central stele; db, dorsal bundle; lbc, lateral bundle complex; ob, ovule bundle; pcm, parenchyma; pus, pseudostele; st, stamen; stb, stamen bundle; vb, ventral bundle. Scale bars = 500 μm.

*A. luzonensis* usually exhibits two to four completely separate carpels (Figure 1A,G). The carpel primordia are almost hemispherically initiated and larger than the stamen primordia (Figure 1F). Each carpel consists of a plicate zone, a very short ascidiate zone (Figure 3G, Figure 5I,J), and a long, conspicuous stipe (Figure 2F). Carpel stipe ontogenesis occurs at the early stages of carpel development (Figure 2B). The continuous growth of the flanks on the ventral side of the young carpel triggers its early closure; however, the closure does not extend to the base of the carpel, where the carpel stipe was previously present (Figure 2C). Subsequently, the dorsal region of each carpel thickens markedly (Figure 2D), and the stigma forms (Figure 2E). At anthesis, the carpels are the widest at the basal region, with an arch on the abaxial side. The carpel stipe remains elongated, accounting for approximately a quarter of the carpel length at anthesis, and continues to elongate during the fruiting stage (Figure 2F). Each carpel has two lateral ovules with the placentae at the ovary base (Figure 3H, Figure 5L). 

*A. Javanica* exhibits a multicarpellate gynoecium (Figure 1B,J). The carpels are completely separate and appear whorled at initiation (Figure 1I); as the carpel volume increases, the whorled structure becomes less obvious because the space in the floral apex becomes limited. Each carpel consists of a plicate zone and a conspicuous carpel stipe (Figure 2J) but lacks the short ascidiate zone. Carpel stipe ontogenesis occurs in the early stages of carpel development (Figure 2H) and remains elongated during the flowering and fruiting stages (Figure 1D, Figure 2I,J). Each carpel has two anatropous ovules, with the placentae at the lateral side of the ovary base. The ovules ascend and the micropyle turns downward with the development of the ovule. The micropyle is formed by the inner integuments (Figure 2K–O).

### 2.2. Vasculature from Receptacle to Carpel

**Figure A1:**
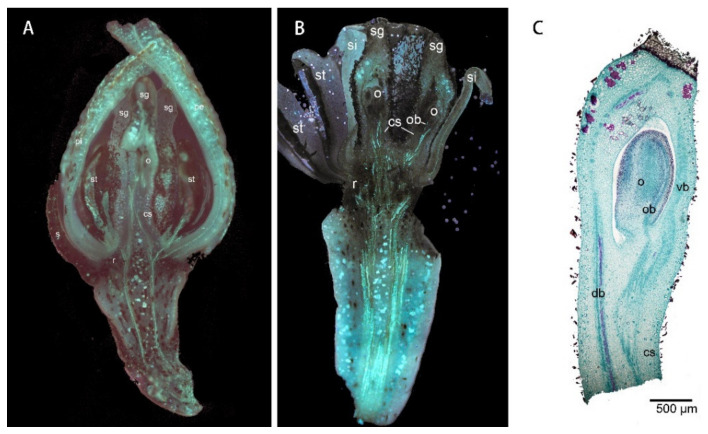
Longitudinal sections of *Anaxagorea* flower and carpel. (**A**) Longitudinal section of the flower of *Anaxagorea luzonensis*. (**B**) Longitudinal section of the flower (without perianth) of *Anaxagorea javanica*. (**C**) Longitudinal section of the carpel of *A. javanica*. Db, dorsal bundle; cs, carpel stipe; o, ovule; ob, ovule bundle; pe, outer petal; pi, inner petal; r, receptacle; s, sepal; st, stamen; si, staminode; sg, stigma; vb, ventral bundle.

**Figure A2:**
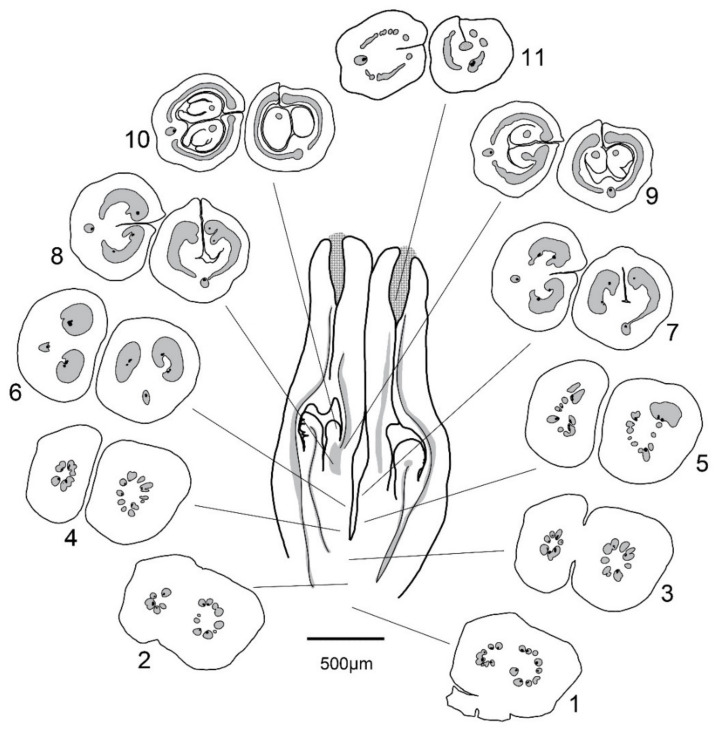
Carpel architecture of *Anaxagorea luzonensis* in flower stage – longitudinal section with related ascending transverse sections.

In the *A. luzonensis* cross-sections, the receptacle base presents a hexagon of 18 bundles from the pedicel stele (Figure 3A). The hexagon has six breaks, which build up a crown of the cortical vascular system to supply the sepals and the two whorls of petals and the stamens (Figure 3B, Figure A1A). The central stele, composed of 18 bundles, finally breaks into two nine-bundle groups at the floral apex and run into the two carpel gynoecium (Figure 3C,D). Each group of nine bundles assembles as a basal ring around the parenchyma at each carpel base (Figure 3E). At the slightly upper part of each carpel, several bundles emerge on the lateral side, and the basal ring breaks, from which the dorsal bundle separates and the lateral bundles reorganize into two groups of lateral bundle complexes (Figure 3F). In each of the lateral bundle complexes, the adjacent bundles tend to join, assembling into a concentric pattern (the phloem surrounds the xylem) (Figure 3G). Below each placenta, each of the concentric lateral bundle complexes transform into a set of “C”-shaped lateral bundle complexes, from which the ovule bundles separate, while the other bundles run into the ovary wall. There are no horizontal connections between the dorsal and the other bundles (Figure 3H). The corresponding positional relationship between ascending transverse sections of the carpels and longitudinal section of the carpels is shown in (Figure A2).

**Figure 4:**
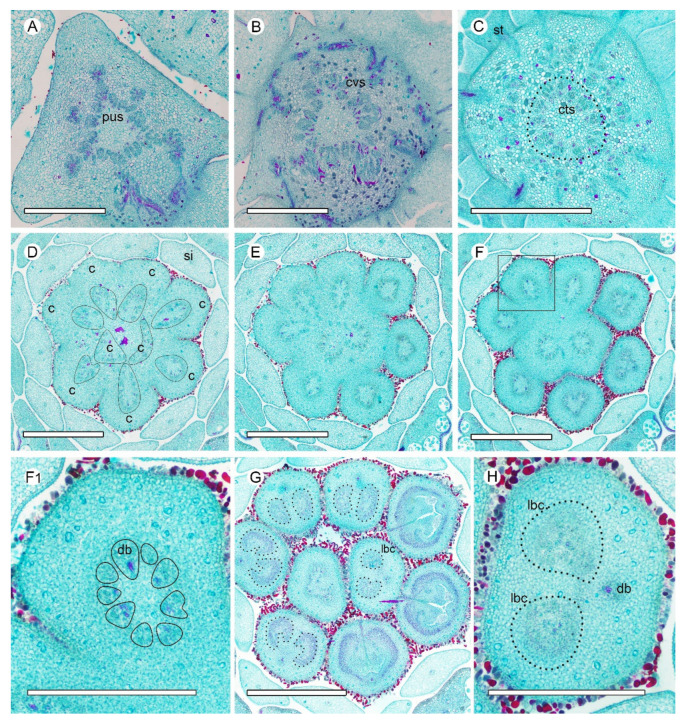
Ascending paraffin transections of *Anaxagorea javanica* flower. (**A**) Base of receptacle, showing six groups of vascular bundles and sepal connections. (**B**) Points of petal connection to receptacle, showing perianth bundles. (**C**) Androecial bundles serving stamens by repeated branching. (**D**,**E**) Base of gynoecium, showing enlarged central stele breaks and bundles distributed into carpels (bundles of each carpel is marked by a solid line). (**F**,**G**) Carpel vasculature at different positions. (**F1**) Detailed view of a carpel in **F** (marked with a box), showing vascular bundles making up the basal ring (each bundle is marked by a solid line). (**H**) Concentric lateral bundle complexes in carpel. C, carpel; cts, central stele; cvs, cortical vascular system; db, dorsal bundle; lbc, lateral bundle complex; pus, pseudostele; si, staminode; st, stamen. Scale bars = 500 μm.

The pseudostele at the base of the *A. Javanica* receptacle is triangular, with ~45 bundles. The outer six cortical traces are cylindrical and serve the sepals and petals (Figure 4A,B). At a slightly higher level, the androecial bundles emerge and served the stamens by repeated branching, and the staminode bundles emerge as a crown around the central stele (Figure 4C, Figure A1B). Before entering the gynoecium, the central stele enlarges and breaks up into ~70 bundles to supply the nine carpels, and each carpel is served by 7–10 bundles (Figure 4D,E). The vascular bundle arrangement is similar to the ascending sections in *A. luzonensis*, with the basal ring and the concentric lateral bundle complexes presents in each carpel (Figure 4F–H).

#### 2.3. D-Reconstruction of Carpel Vasculature

**Figure 5:**
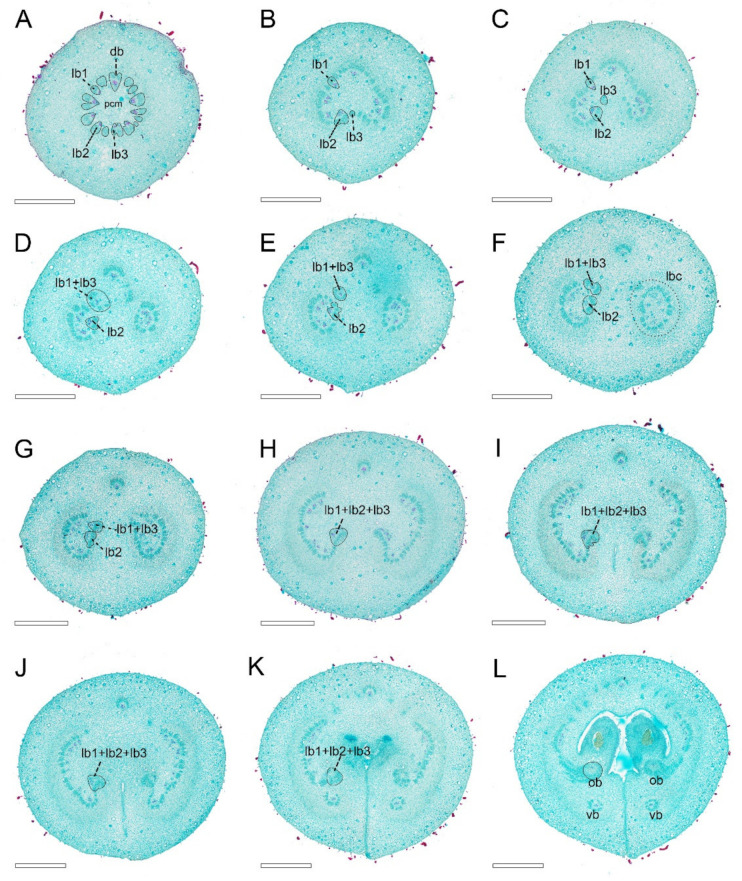
Ascending paraffin transections of mature *Anaxagorea luzonensis* carpel. Vascular bundles associated with one of the ovule bundles are labeled by solid lines in (**B**–**L**). (**A**) Carpel base, showing the basal ring. (**B**,**C**) Basal ring breaks on ventral side. (**D**–**F**) Ascending carpel stipe sections, showing lateral bundles reconstituted to two sets of ring-arranged lateral bundle complexes (labeled by the dotted line). (**G**,**H**) Top of carpel stipe, showing “C”-shaped lateral bundle complex. (**I**–**K**) Below ovary locule, showing formation of ovule bundles. (**L**) Base of ovary locule. Db, dorsal bundle; lb, lateral bundle; lbc, lateral bundle complex; ob, ovule bundle; pcm, parenchyma; vb, ventral bundle. Scale bars = 500 μm.

At the base of a mature *A. luzonensis* carpel, 15 discrete bundles are arranged in a radiosymmetric pattern, forming a basal ring around the central parenchyma (Figure 5A). At the slightly upper part, the basal ring curves inward on the ventral side and breaks away from the invagination (Figure 5B,C). The bundles (except the dorsal) divide into two groups on each side of the carpel, each forming a lateral bundle complex, which was also ring-arranged. The ring-arranged lateral bundle complexes correspond to the above-mentioned sections of the concentric lateral bundle complexes at the flowering stage (Figure 5D–F). Below each placenta, bundles of each lateral bundle complex break up on the dorsal side and transform into a “C”-shaped lateral bundle complex (Figure 5G,H). The bundles on the ventral side of each lateral bundle complex gather together (excluding the ventral bundle) and enters each ovule, while other bundles enter into the ovary wall. The ovule bundles are concentric. (Figure 5I–L). 

**Figure 6:**
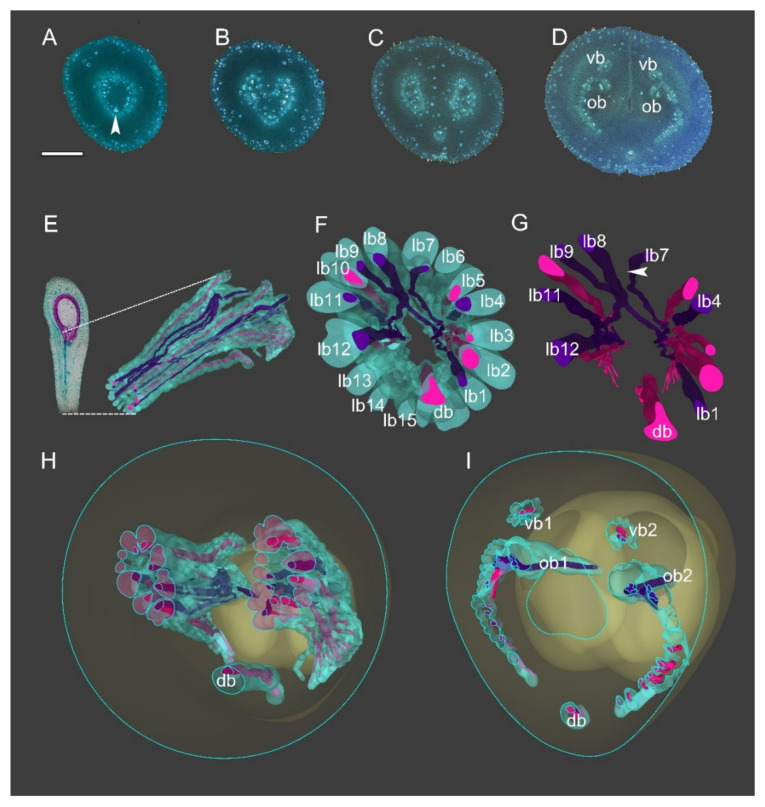
Three-dimensional construction of *Anaxagorea javanica* vasculature. Bundle outlines colored green, xylem red, and purple, among which bundles associated with ovule bundles are colored purple. (**A**–**D**) Aniline blue-stained *A. javanica* sections, selected from the consecutive cross-sections for modeling. (**E**) Longitudinal section of mature *A. javanica* carpel (left) and 3D vasculature model; dotted lines on longitudinal section indicate vasculature position in carpel. (**F**) Perspective from base of carpel vasculature. (**G**) Perspective from base of carpel (xylem only). The arrow indicates the intersection of two lateral bundles which fed two ovules, respectively. (**H**) Cross-section of 3D model corresponding to **C**, showing ring-arranged lateral bundle complexes. (**I**) 3D model section showing distribution of vascular bundles at base of ovary. Db, dorsal bundle; lb, lateral bundle; ob, ovule bundle; vb, ventral bundle. Scale bars = 500 μm.

**Figure A3:**
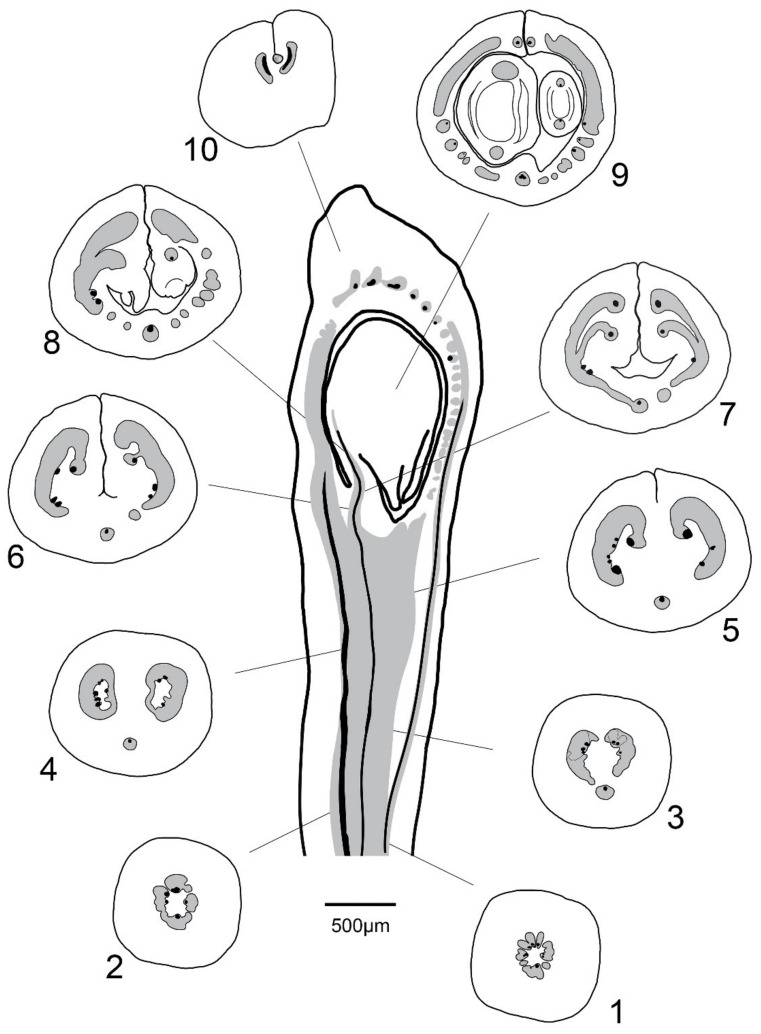
Carpel architecture of *Anaxagorea javanica* in fruit stage – longitudinal section with related ascending transverse sections.

Consecutive cross-sections of *A. Javanica* are similar in vasculature to those of *A. luzonensis* (Figure 6A–D). The base of the mature *A. Javanica* carpel exhibits 16 distinct bundles forming the basal ring (Figure 6A,F). The 3D model shows that (1) the basal ring and lateral bundle complex are cylindrical (Figure 6F,H). (2) The ovules were fed directly by bundles from the base of the carpel through the lateral bundle complex. (3) Each ovule bundle was formed from several non-adjacent lateral bundles, and two of their bundles that fed each ovule joined on the ventral side (Figure 6G,I). (4) The dorsal bundle remained independent throughout ontogenesis (see also Figure A1C), without any link to other bundles (for details, please refer to the Supplementary Materials). The corresponding positional relationship between ascending transverse sections of the carpel and longitudinal section of the carpel is shown in (Figure A3).

## 3. Discussion

Observations of the continuous changes in vasculature from the receptacle to the carpel revealed that (1) all the carpel bundles were only connected with the central stele of the receptacle, (2) vascular bundles at both the carpel stipe and the ovule/placenta are in a radiosymmetrical pattern, (3) the young concentric bundles develop into a ring-arranged bundle complex with carpel maturation, and (4) all the radiosymmetric vasculatures in the carpel were fed by a larger radiosymmetric bundle system.

### 3.1. Carpel Organogenesis

One of the distinctions between *Anaxagorea* and other genera of Annonaceae is that it has a pronounced stipe in the floral stage, unlike the stipe that develops in the fruit stage in other Annonaceae. In some studies, *Anaxagorea* carpels have been reported to exhibit an ascidiate base [28], while they have been described as completely plicate in others [29]. In the present study, floral organogenesis revealed that the carpel stipe emerges from the base of *A. luzonensis* and *A. javanica* carpels in the early stages of carpel development and elongates with the development of the carpel. In the flowering stage, the ventral slit of *A. luzonensis* terminates close to the base of the ovary locule, resulting in a very short ascidiate zone, while in *A. javanica*, it may continue below the ovary locule. Such variations suggest a transformation from semi-ascidiate (i.e., carpels with an ascidiate base) to plicate carpels in the genus.

### 3.2. Carpel Vasculature

Previous studies have reported that the Annonaceae gynoecium is fed by an enlarged central stele, and each carpel is usually fed by three bundles, one median and two lateral [30,31,32,33]. However, in *A. luzonensis* and *A. javanica*, the number of vascular bundles that fed the carpel during anthesis is significantly more than three, regardless of the number of carpels, and the number of vascular bundles enter the *A. luzonensis* gynoecium is consistent with the central stele. The bundles entering the carpel are arranged in a radiosymmetric pattern, and this pattern maintains spatiotemporal continuity throughout the carpel stipe. In the basal ring, there are two lateral bundles that are fed to both ovules bond together (lb8 and lb9 in Figure 6G), which makes the topological structure of the basal ring unable to be flattened into a leaf-like structure bearing marginal ovules. 

It has been reported that in *Anaxagorea*, the ovules are served by the lateral bundle complex from the base of the carpel [29,34]. This pattern is different from most cases in Annonaceae, in which ovules are served by separate vascular bundles branching directly from the dorsal bundles, such as in *Cananga*, *Deeringothamnus*, and *Pseudartabotrys* [32,33,35], or from relatively dorsally positioned bundles of the lateral bundle networks, such as in *Meiocarpidium* and *Ambavia* [35,36]. Observations of the different developmental stages of the *Anaxagorea* carpel revealed that the bundles of the lateral bundle complexes are also arranged in a ring, and each of the lateral bundle complexes developed from a young concentric bundle. The 3D model showed that the ring-arranged lateral bundle complexes play a key role in forming the ovule bundles because it facilitates the approaching and merging of non-adjacent bundles. The ovule bundles are also concentric. The dorsal bundle remained independent throughout, and there were no horizontal connections between the dorsal bundle and the lateral bundle complexes. The ventral bundle participated in the formation of the spatial ring-arrangement of the lateral bundle complexes; however, it was not involved in the formation of ovule bundles. 

### 3.3. Implications for Evolution

Endress preferred the description that the ovule was once not associated with the carpel and that the association was rooted at the earliest stage of the evolution of angiosperms. He also emphasized that the carpel primordium has a certain volume and most of this volume is fixed inside the floral apex [37]. The floral apex is the primordium of a flower or of the inner part of a flower, from which everything (floral organs) develops [37,38,39,40]. Recent studies have shown that the ovule in angiosperms is a branch-leaf complex, which originates from the uniovulate cupule shoot [12,41,42]. Thereafter, when the foliar structure (carpel wall) is involved, the ovule needs to be connected to the flower apex through the carpel. Since the ovule is equivalent to a shoot, the carpel that connects the flower apex and the ovule cannot be a phyllome, which is further supported by the repeatedly presented radiosymmetric vasculature pattern in the carpel of *Anaxagorea*. The formation of floral organ vasculature is initiated by the activity of meristem parts of the organ primordium within the floral apex [43,44]. In *A. luzonensis* and *A. javanica*, the vasculature pattern entering the carpel from the central stele was considerably distinct from that entering the perianth. With the development of the carpel, the ovule bundle, the lateral bundle complex, the basal ring, and the central stele increasingly exhibit similar radiosymmetric patterns, which are also similar to those of shoots.

Recently published materials from the Inner Mongolia material that show the bract on the seed-bearing unit suggest that the carpel evolved by synorganization involving a recurved uniovulate cupule and a subtending bract or leaf [12], i.e., the carpel originates from a shortened, simplified, and stopped shoot. Current research shows that the recruitment of the meristem termination and organ identity genes *CRC* and *SEP1-*4 coincides with the origin of angiosperms [45,46,47]. The atypical carpel development in *Michelia* and the ontogeny of *Illicium* also suggests that the ovule is an axillary branch to the foliar part of the carpel, and gradually shifts from the axilla to the carpel wall [18,19]. The usual definitive ovule arrangement is on the adaxial side of the carpel, as a result of the megasporophore base immersion in the carpel matrix, not in its axil [48]. The anatomical result of the present study supports the above interpretation that the two sets of ovule bundles were fed by the central stele through the ring-arranged bundles of the carpel stipe, but not to the ovary wall. In addition, the two ovule bundles are connected, despite the two ovules developing on each flank of the carpel.

## 4. Materials and Methods

### 4.1. Scanning Electron Microscopy and Paraffin Sectioning

*A. luzonensis* flower samples at different floral stages (from early bud to young fruit) were collected from the Diaoluo Mountain, Hainan, China, in July 2017 and *A. javanica* from the Xishuangbanna Tropical Botanical Garden, Yunnan, China in May 2017. The gynoecia were isolated and preserved in 70% formalin-acetic acid-alcohol (5:5:90, *v*/*v*), and the fixed specimens were dehydrated in a 50% to 100% alcohol series. To delineate the structure and development of the carpel, carpels were removed from the gynoecia, passed through a 50% to 100% iso-pentanol acetate series (SCR, Shanghai, China), critically point-dried, sputter-coated with gold, observed, and photographed under a scanning electron microscope (Tescan VEGA-3-LMU, Brno, Czech Republic). Flowers and carpels were embedded in paraffin, serially sectioned into 10–12-µm thick sections, and stained with Safranin O and Fast Green to illustrate the vasculature. The transverse sections were examined and photographed using a bright-field microscope (Olympus BX-43-U, Tokyo, Japan). In addition, longitudinal hand-cut sections were made and observed for a rough check and better understanding of the vasculature.

### 4.2. Topological Analysis of Carpel Vasculature

Consecutive paraffin sections (12 µm each and 423 sections total) of *A. javanica* were stained with aniline blue, examined, and photographed after excitation at 365 nm using an epifluorescence microscope (Olympus BX-43-U, Tokyo, Japan) and a semiconductor refrigeration-charged coupled device (RisingCam, MTR3CMOS). Manual image calibration of graphic edges was carried out using Photoshop CC 2017 (Adobe, San Jose, CA, USA). Forty-five images were selected equidistantly (except the last 5 intervals—their distance is half that of the others, because the structure changes greatly) for 3D reconstruction from the 423 section images obtained. The figures were organized according to the vascular bundle outlines of the sections using Photoshop CC 2017 (Adobe) and Illustrator CC 2017 (Adobe). The xylem and phloem contours were manually drawn, extracted as paths with a pen tool, and exported in DWG format. The DWG files were imported into 3Ds Max 2016 (Autodesk, San Rafael, CA, USA) and sorted according to the distances and orders of the sections. The paths were converted into Editable Spline curves to generate the basic modeling contour. The Loft command of Compound Objects was used to obtain the shape of the Editable Spline, and a complete 3D carpel vasculature model was generated.

## 5. Conclusions

In the present study, 3D reconstruction was used to resolve the complex spatial carpel vasculature relationship in *Anaxagorea*. This allows the identification of the carpel vascular bundles associated with the formation of ovule bundles. According to the results, the radiosymmetric vasculature patterns in the carpel of *Anaxagorea* are repeatedly presented in the pedicel, the receptacle, the base of the carpel, and the placenta. Each is fed by a larger radiosymmetric bundle system, providing anatomical evidence of the composite origin of the carpel.

The hypothesis that the carpel evolved from synorganization of a female reproductive shoot and a subtending bract or leaf has been supported by various pieces of evidence from fossils, molecular biology, development, and, now, anatomy. More evidence from basal angiosperms would be required to demonstrate whether the radiosymmetric pattern is primitive or derived. If the pattern is common in angiosperms, it could be taken into account in future morphological cladistic analyses for seed plants.

## Supplementary Materials

The following are available online at https://www.mdpi.com/article/10.3390/plants10102221/s1, Video S1: 3D Reconstruction of Anaxagorea Carpel Vasculature.
